# Recent Progress in Enhancing Fungal Disease Resistance in Ornamental Plants

**DOI:** 10.3390/ijms22157956

**Published:** 2021-07-26

**Authors:** Manjulatha Mekapogu, Jae-A Jung, Oh-Keun Kwon, Myung-Suk Ahn, Hyun-Young Song, Seonghoe Jang

**Affiliations:** 1 Floriculture Research Division, National Institute of Horticultural and Herbal Science, Rural Development Administration, Wanju 55365, Korea; manjubio7@gmail.com (M.M.); kok5510@korea.kr (O.-K.K.); ahnms@korea.kr (M.-S.A.); shy0817@korea.kr (H.-Y.S.); 2 World Vegetable Center Korea Office (WKO), Wanju 55365, Korea

**Keywords:** fungal diseases, genetic engineering, HIGS (host-induced gene silencing), SIGS (spray-induced gene silencing), ornamental plants, resistance mechanisms, breeding technology, *Botrytis cinerea*, *Fusarium oxysporum*, *Alternaria* sp.

## Abstract

Fungal diseases pose a major threat to ornamental plants, with an increasing percentage of pathogen-driven host losses. In ornamental plants, management of the majority of fungal diseases primarily depends upon chemical control methods that are often non-specific. Host basal resistance, which is deficient in many ornamental plants, plays a key role in combating diseases. Despite their economic importance, conventional and molecular breeding approaches in ornamental plants to facilitate disease resistance are lagging, and this is predominantly due to their complex genomes, limited availability of gene pools, and degree of heterozygosity. Although genetic engineering in ornamental plants offers feasible methods to overcome the intrinsic barriers of classical breeding, achievements have mainly been reported only in regard to the modification of floral attributes in ornamentals. The unavailability of transformation protocols and candidate gene resources for several ornamental crops presents an obstacle for tackling the functional studies on disease resistance. Recently, multiomics technologies, in combination with genome editing tools, have provided shortcuts to examine the molecular and genetic regulatory mechanisms underlying fungal disease resistance, ultimately leading to the subsequent advances in the development of novel cultivars with desired fungal disease-resistant traits, in ornamental crops. Although fungal diseases constitute the majority of ornamental plant diseases, a comprehensive overview of this highly important fungal disease resistance seems to be insufficient in the field of ornamental horticulture. Hence, in this review, we highlight the representative mechanisms of the fungal infection-related resistance to pathogens in plants, with a focus on ornamental crops. Recent progress in molecular breeding, genetic engineering strategies, and RNAi technologies, such as HIGS and SIGS for the enhancement of fungal disease resistance in various important ornamental crops, is also described.

## 1. Introduction

Ornamental plants possess natural beauty and are distinctive due to their exquisite blooms. The alluring colors and shapes of their flowers, leaves, and fruits of these plants are a source of major attraction. Ornamental crops are grown for various decorative purposes as potted plants, woody ornamentals, cut flowers or cut foliage, bulbs, and corms [1]. The floriculture sector is flourishing globally and is experiencing increased demand. Floriculture has significantly impacted the horticultural industry by facilitating a substantial turnover with regard to all aspects of floriculture, of which roughly one third of the global value of the ornamental market is made up of cut flowers [2]. The turnover of popular ornamental plants in the world’s largest flower auction, the Royal FloraHolland auction is detailed in Table 1 [3] (FloraHolland Key figures, 2019). As this represents a dynamic sector, introducing novelties into the market is a mandate for withstanding global competitiveness.

Plant pathogens cause severe losses in the production and/or quality of various ornamental crops and this is of great economic significance. Their effects range from mild symptoms to catastrophes, where larger areas of planted crops are seriously damaged [4]. Ornamental plants in general are infected by a myriad of microbial organisms, including bacteria, fungi, and viruses, that severely affect the growth and morphology of these plants and thereby influence their commercial value. The visual quality of ornamental plants is critical, particularly for cut flowers and potted plants. Visual disease symptoms and the impact on growth caused by pathogens both heavily affect the quality and the market value of the flowers. Hence, ensuring quality traits in these plants is essential as they face increasing demand for industrial purposes [5].

Among the diseases caused by fungi, bacteria, viruses, and viroids, approximately 70% of those in plants are caused by fungi. In many cases, fungal diseases cause a significant reduction in crop quality and yield that can represent up to 30–40% of the total potential yield [6]. Fungi are estimated to be the biggest threat and the major cause underlying pathogen-driven host losses, declining the visual quality and lowering of market prices of ornamental flowers [7]. The majority of plant fungi are strictly saprophytic and derive their nutrition from dead organic matter, while the remaining are pathogenic biotrophic and necrotropic fungi that grow on living plants and cause diseases [8]. Plant fungal pathogens can be largely classified into the phyla Ascomycota and Basidiomycota. Ascomycetes are represented by various classes of pathogens such as Sordariomycetes (*Magnaporthe* spp.), Dothideomycetes (*Cladosporium* spp.), and Leotiomycetes (*Botrytis* spp.), while Basidiomycetes includes two larger groups of plant pathogens such as the rusts (Pucciniomycetes) and the smuts (*Ustilaginomycetes*). Based on the nature of their interaction with plants, these pathogenic fungi are grouped into biotrophs that form an intimate interaction with the host plant and utilize its living tissues and the necrotrophs that kill the plant tissues by causing cellular necrosis that eventually leads to plant death [9]. Obligate biotrophic fungi cannot grow without a living host and cause various diseases in ornamental plants such as leaf spots, blights, rusts, smuts, powdery mildew disease, and downy mildew disease. 

Frequently, fungal diseases are managed by the application of chemical fungicides that are effective only for a few diseases and are sometimes non-specific. Moreover, excessive use of chemicals results in pathogen resistance against these chemicals and is highly undesirable due to health and environmental safety concerns [10]. An alternative method to chemical control is the biological control of pathogens, and this can be achieved through an integrated approach for disease management [1]. However, the scope of disease control provided by biocontrol methods is very limited. The formulations of beneficial fungi or bacteria that suppress plant pathogen growth usually provide some degree of control and can only be used as a component of the IPM strategy [11]. Hence, the ultimate goal is to generate plants that possess increased resistance to diseases. Effective control of diseases can be achieved by host basal resistance, as this can reduce the requirements of pesticide application. However, not all ornamental plants possess natural disease resistance; therefore, disease management relies on the use of disease-resistant varieties. Hence, it is important to elucidate pathogenicity and host-pathogen interactions to develop novel strategies for improving disease resistance in plants [12]. The development of disease-resistant varieties is possible via traditional breeding approaches or genetic engineering by introducing resistance mechanisms derived from other plant species or pathogens [13]. The introduction of natural resistance by traditional breeding approaches includes non-transgenic breeding programs, such as DNA-based marker-assisted selection that may require several cycles of breeding to combine the disease-resistant trait and desirable ornamental characteristics into a single plant genotype. In contrast, the transgenic approach uses tightly regulated transgenes to introduce specific or broad-spectrum disease resistance into genotypes with elite ornamental qualities [14]. Breeding efforts to achieve disease resistance in ornamental plants are comparatively limited, as the disease-resistance trait is typically taken into consideration only during the later stages of the breeding line selection process of cultivar development [15]. Genetic mapping of disease resistance is relatively scarce due to the large and complex genomes and the nature of the polyploidy present in most ornamental plants, as these characteristics require a greater number of resources and more time to map the resistance mechanisms [16]. Nevertheless, recent advances in genome-sequencing technologies, phenotyping, marker development, and genotyping have provided a promising base for further breeding development for disease resistance in ornamental plants. Alternatively, genetic engineering technology provides a potential platform for the improvement of resistance to a myriad of biotic and abiotic stresses in ornamental plants, thus improving plant quality. Tolerance to several fungal diseases has been achieved by transferring various genes such as *glucanase*, *chitinase*, *defensin*, *osmotin*, and *pathogenesis-related* (*PR*) genes into ornamental plants [17]. RNA interference (RNAi) strategies have also demonstrated the potential to protect plants against pathogens, and one of these strategies is host-induced gene silencing (HIGS), mediated by RNAi signals generated in planta [18]. In addition to HIGS, a novel strategy designated as ‘spray-induced gene silencing’ (SIGS) has been demonstrated to protect plants from fungal pathogens through the direct spraying of dsRNA-targeting pathogen genes in plant tissues, thus displaying the potential to be used as an alternative to conventional fungicides [19]. Furthermore, advances in genome editing technology and its applications have offered greater possibilities with regard to precise manipulation of the genome sequences at genes of interest, and these techniques are currently being used to improve disease resistance in plants [20]. Research advances, such as biotechnological advancements used for improving resistance against fungal diseases in some economically important ornamentals such as the rose, chrysanthemum, gerbera, lily, carnation, and petunia, are discussed in the current review. 

## 2. General Mechanisms of Fungal Pathogen Infection and Plant Resistance

**Figure 1:**
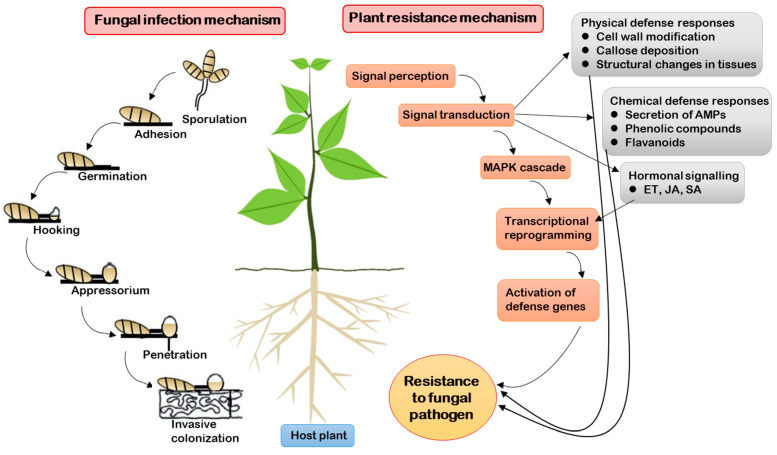
Schematic representation of the various steps involved in the fungal infection process and the general mechanism of the defense response of the host plant machinery to combat the fungal pathogen and to provide resistance.

Various fungi can infect different parts of plants, including the aerial and the underground parts. Typically, many fungal pathogens exhibit organ specificity and do not attack all parts of the host plant. The general establishment of fungal infection and pathogenicity involves the following steps: (a) adhesion to the plant surface, (b) germination on the plant surface and formation of infection structures, (c) penetration of the host, and (d) colonization of the host tissue [21]. For the initial step of fungal attachment to the plant surface, fungi utilize different mechanisms for different organs and roots due to variations in the surface hydrophobicities of these structures. Secretion of extracellular enzymes from spores is required for adhesion to the plant surface. The aerial parts of the plant are protected with a cuticle that includes pectin, cutin, and wax layers, and the cutin-degrading enzyme cutinase is associated with fungal pathogenicity [22]. Adhesion of spores to host surfaces requires spore-tip mucilage that is released upon the hydration of conidia from the periplasmic compartment of the conidial apex, ultimately resulting in spore-wall rupture [23]. The second step of fungal pathogenicity is spore germination and filamentous germ tube formation, a process that involves complete cell reprogramming and requires specific regulatory networks. After the spore germinates, it produces a germ tube that differentiates into an appressorium. Previous reports have suggested that a G-protein-coupled receptor (Pth11) and cognate G-α- and G-βγ-subunit proteins are required for appressorium development on hydrophobic surfaces [24,25]. The appressorium in turn produces an infection peg and applies a turgor-driven mechanical pressure of approximately 8.0 MPa onto the peg that then pierces the cuticle and grows into the plant’s underlying epidermal cell [26]. The infectious hypha then branches into the secondary hyphae that spread inter- and intra-cellularly within the host tissue [21]. The general mechanism of fungal infection is presented in Figure 1.

Plants are equipped with multilayered defense mechanisms to fight pathogenic microorganisms [27]. They depend upon the ability of their immune system to identify the pathogen, activate signal transduction, and perform defense responses through pathways that involve numerous genes [28]. Plants have evolved to obtain resistance against pathogens by preventing pathogens from gaining access to the cell through the use of physiological barriers and surface-recognition features [29,30]. After sensing the pathogen, the plant’s trans-membrane receptors and intracellular receptors activate defense systems, either by physically interacting with pathogen-derived immunogens or by examining the host-target modifications induced by the pathogen [31,32]. Furthermore, plant-derived antimicrobial peptides, that are produced in response to infection, function to suppress pathogenicity by facilitating direct detoxification or by inhibiting the activity of pathogenic factors [33,34]. Pattern-recognition receptors (PRRs) play a key role in plant defense mechanisms [35]. PRRs recognize pathogen-associated molecular patterns (PAMPs) and eventually initiate PAMP-triggered immunity (PTI) that functions as a specific plant defense response against pathogens [28,36]. However, plants often experience effector-triggered susceptibility (ETS) where several pathogens secrete effectors to defend the plant PTI and suppress it by activating susceptibility (S) proteins, ultimately progressing the infection [35]. Subsequently, the second line of defense in plants is activated to respond to these effectors by recruiting resistance (R) genes. Signals from effector molecules activate R genes, thus leading to effector-triggered immunity (ETI) [37]. Both PTI and ETI result in the induction of pathogenesis-related (PR) genes, and the activation of mitogen-activated protein kinase (MAPK) cascades that function as important signal transducers to channel information through protein phosphorylation/dephosphorylation processes [38]. Furthermore, MAPKs promote defense systems that threaten the survival of fungal cells by inducing downstream responses, including hypersensitive response (HR), cell-wall modification, callose deposition at the site of infection, and secretion of antimicrobial proteins (AMPs) such as chitinases, defensins, phytoalexins, and protease inhibitors [28,39]. In addition to these plant immune responses, the host plant may induce immune responses that are specific to the infecting fungal culture [40,41]. Plant hormones play a significant role in signal transduction during plant defense. The salicylic acid (SA) pathway is stimulated during the defense response against biotrophic and hemibiotrophic pathogens, while primary hormones such as jasmonate (JA) and ethylene (ET) are involved in the defense response against necrotrophic pathogens [42]. The general mechanism of plant resistance to fungal pathogens is presented in Figure 1.

## 3. Molecular Breeding for Fungal Disease Resistance

Despite the rapidly growing economic importance of ornamental crops, the breeding strategies used for developing new cultivars which possess disease resistance, lag behind those of other agricultural crops. Complex genomes, limited availability of gene pools, and a lack of genetic variability within these gene pools are major limitations in the use of conventional breeding to acquire disease resistance in ornamental plants. However, relentless efforts to map disease resistance traits and to facilitate the introgression of disease resistance genes into superior ornamental plants, have been put forth in combination with recent technological progress in genome sequencing, genotyping, and the development of markers. Among the molecular tools used for efficient breeding, marker-assisted breeding (MAB) approaches, which use trait-linked molecular markers identified from genetic, genomic, and molecular studies, have enabled the development of more effective selection strategies [43]. In particular, the MAB for disease-resistance traits, that generally require simultaneous combinations of multiple genes, has been deployed to enhance efficiency. Moreover, MAB may efficiently facilitate target-gene introgression from the wild species genomes against the genetic background of wild donors [44]. Ornamental crops possess the desired set of traits for floral characteristics such as floral color, shape, and size. Therefore, making ornamental plants resistant to diseases in addition to possessing these floral traits is required for hundreds of cultivars [45].

In the rose plant (*Rosa hybrida*), several markers, such as amplified fragment length polymorphisms (AFLPs), restriction fragment length polymorphisms (RFLPs), simple sequence repeats (SSRs), cleaved amplified polymorphic sequences (CAPS), sequence-characterized amplified regions (SCARs), and various other markers have been developed and subsequently utilized to create diploid and tetraploid maps of roses [46,47,48]. Loci for a number of disease-resistant traits have been determined from those maps, and three *Rdr* genes have also been localized on the rose genome. Additionally, their quantitative inheritance pattern has been analyzed for black spots [49]. Linde et al. (2004) mapped a dominant-resistance gene (*Rpp1*) that exhibited race-specific resistance to powdery mildew in roses by utilizing newly developed SCAR markers [50]. Several quantitative trait loci (QTLs) specific for resistance to powdery mildew have also been reported in roses [51,52,53]. Four markers for two QTLs have been reported in *Rosa roxburghii* for powdery mildew resistance; three of these are associated with *Rdr1*, while the last is associated with *Rdr3* [54,55]. Additionally, Terefe-Ayana et al. (2011) reported the molecular characterization of the dominant resistance gene *Rdr1,* against one isolate of the leaf spot pathogen *Diplocarpon rosae* in roses [56]. Recently, four *MLO* genes that serve as candidate genes for powdery mildew resistance have been mapped to three linkage groups in roses [57].

Although traditional breeding has led to the development of numerous *chrysanthemum* cultivars as one of the most popular ornamental plants, the application of molecular breeding for fungal disease resistance is still lagging compared to that of other crops, and this is primarily due to the complex genome allohexaploidy, heterozygosity, self-incompatibility, and the inbreeding depression of this plant [43]. Thus, approaches using genetic mapping, genome-wide association studies (GWAS), and molecular markers associated with traits in *chrysanthemum* have been limited [58]. An AFLP marker linked to white rust resistance was developed using bulk segregant analysis (BSA). This AFLP marker was further converted into an SCAR marker that was confirmed in the pseudo-F1 test cross [59]. Hence, this marker can be successfully used to screen white rust-resistant populations in chrysanthemum breeding programs. Genome sequence information for a diploid wild *Chrysanthemum seticuspe* will help to accelerate the molecular breeding of *chrysanthemum* for disease resistance [60].

The lily (*Lillium*) is the most economically important monocot bulbaceous flower. However, breeding for disease resistance in lilies is hindered due to their long juvenile phase that limits the selection process for the introgression of desired traits into a single cultivar. Significant QTL mapping for *Fusarium* resistance in lilies was conducted, and the results demonstrated the mapping of four QTLs to linkage groups 1, 5, 13, and 16 of the AFLP genetic map [61]. Among these four QTLs, the most tightly linked markers to the two significant QTLs for *Fusarium* resistance were converted into robust PCR-based markers that can be successfully used in molecular breeding in lilies [62]. Later, Shahin et al. (2011) remapped major genes for *Fusarium* disease resistance in lilies using three different molecular marker systems (AFLP, DArT, and NBS profiling), thus resulting in the identification of two additional QTLs for *Fusarium* resistance [63]. Recently, high-quality transcriptomic analyses of lilies and tulips have become available and these offer valuable information for use in the genomic studies of bulbous plants [64]. Tang et al. (2015) constructed a genetic linkage map for *Fusarium* resistance in tulips using a combined set of AFLP, NBS, and SSR markers, thus leading to the identification of six putative QTLs. Of these, three were well-matched to the phenotype and could be used for marker-assisted selection (MAS) in tulip breeding [65]. 

*Gerbera hybrida* is a commercially predominant and popular cut-flower crop that is also a highly heterozygous ornamental plant. To date, only a limited number of resistance gene analogs (RGAs) and SSR markers have been available for genetic studies in gerbera. However, reliable single-nucleotide polymorphism (SNP) markers have been identified from the transcriptomic analyses of four gerbera genotypes, and these can be further used in the mapping and genetic studies of gerbera. This study also predicted the candidate genes that were related to the biosynthetic pathways of ethylene and jasmonate and the signaling networks that are considered to play a role in plant resistance against *Botryitis cinerea* [66]. The SNP markers identified in the study were further used to construct the first genetic linkage map in gerbera, and a QTL map was developed for resistance to *B. cinera*. A total of 20 QTLs were identified, thus reflecting the complex mechanism of the defense response of this plant against *B. cinera* [67].

The carnation (*Dianthus caryophyllus*) is one of the major ornamental cut flowers worldwide. The carnation is diploid and its genome size is relatively small, compared to that of other ornamental crops. To contribute to molecular and genetic studies, transcriptome analysis was performed in the carnation and the resulting data revealed 17,362 potential SSRs and 14,291 unigenes [68]. Yagi et al. (2013) constructed a high-density genetic linkage map based on SSR markers using RNA-sequence analyses [69]. This genetic linkage map combined with SSR markers serves as a reference genetic linkage map for the members of *Dianthus*, including the carnation, and it can be further used for the mapping of QTLs that are associated with the disease-resistant traits in carnation-breeding programs to develop improved varieties. Recently, the whole-genome sequence of the carnation (*Dianthus caryophyllus* L. cv. Francesco) was determined, ultimately revealing various genes related to disease resistance, floral scent, color, and different metabolic pathways. Additionally, this information served as a resource for fundamental and applied research on carnations, particularly with regard to developing new carnation varieties through molecular breeding approaches [70].

## 4. Genetic Engineering for Improved Fungal Disease Resistance in Ornamental Plants

Although various fungal diseases are managed by fungicide application, fungicides are often non-specific and kill beneficial microbes along with pathogens. Moreover, most fungicides are hazardous chemicals to both human and environmental health. Additionally, excessive use of these chemicals can cause resistance to fungicides [71]. Hence, the development of fungal disease-resistant cultivars would provide a promising alternative method for efficient ornamental production with minimal losses by fungal pathogens. The development of fungal disease resistance through conventional breeding is hindered by several limitations such as deficiency of gene resources for many diseases, the transfer of undesirable traits along with resistant genes, and the rapid evolution of the ability of pathogens to overcome plant-resistance mechanisms [72]. Alternatively, genetic engineering possesses the potential to overcome the barriers in traditional breeding methods and to control the ability of the plant to identify and defend itself against fungal diseases. Advances in genetic engineering have enabled researchers to better understand the molecular mechanisms of plant defense responses, thus contributing to the development of novel strategies to combat the disease [6]. In contrast to conventional breeding, genetic engineering offers the possibility of increasing the disease resistance to several pathogens simultaneously, and the gene of interest can be introduced into the target plant even if the gene does not exist in the natural gene pool [2]. Advances in genetic engineering to achieve fungal disease resistance in various ornamental plants are discussed in this section.

### 4.1. Rose

Roses (*Rosa hybrida*) are cultivated throughout the world and are an economically important ornamental plant worldwide. Roses are most admired for their beauty and fragrance, and they exhibit alluring colors. Within the *Rosa* genus, there are more than 200 rose species and over 30,000 cultivars. They are used as cut flowers, pot plants, and garden plants [73]. Rose-petal essential oils consist of beneficial secondary metabolites that are used in the natural medicine, cosmetics, and perfume industries [74]. However, rose cultivation is severely impaired by major fungal diseases such as powdery mildew, black spots, botrytis blight, downy mildew, and rust that adversely affect yields and product quality [75]. Despite the economic importance of the rose as an ornamental crop, breeding progress for fungal resistance is lagging in roses due to insufficient information regarding disease-resistant traits. Moreover, a higher level of heterozygosity, sterility, and polyploidy are the major limitations of traditional breeding for fungal disease resistance in roses [76]. Hence, genetic engineering is a desirable approach to induce resistance against fungal diseases. Powdery mildew caused by the obligate ascomycete pathogen *Podosphaera pannosa* (Wallr.: Fr.) is one of the predominant fungal diseases of rose. It causes distortion and senescence of the leaves and shoots. Approximately 40% of the fungicide sprayed on cut and potted roses is used to control powdery mildew [77]. It is known that PR genes, including *β-1,3-glucanase*, *chitinase*, *ribosome-inactivating protein* (*RIP*), and *cysteine-rich antimicrobial protein* (*AMP*), are triggered during fungal pathogen infections [78,79]. These antifungal proteins, including chitinases, glucanases, RIPs, plant defensins, and proteinase inhibitors, function by disrupting or suppressing the synthesis of the fungal cell wall. Some of these proteins interact with potential intracellular targets and the plasma membrane of fungi, thus leading to changes in membrane potential and cell death [80]. Plant defensins, including AMPs, are known to interact with glucosylceramides within fungal membranes to induce membrane permeabilization, ultimately leading to fungal cell death [81]. An antimicrobial protein gene (*Ace-AMP1*) isolated from onion seeds that possessed higher plant pathogenic inhibition activity, was introduced into the *Rosa hybrida* cv. Carefree Beauty. The transgenic rose, overexpressing the *Ace-AMP1* gene, was developed to induce fungal disease resistance, and the rose showed enhanced resistance to powdery mildew disease [82]. Furthermore, the transgenic rose, overexpressing antifungal genes such as *class II chitinase* and *type I ribosome inhibiting protein* (*RIP*), exhibited reduced susceptibility to fungal diseases [75]. Transgenic rose plants possessing a high level of expression of the rice *chitinase* gene displayed improved resistance to powdery mildew [83]. Previous studies suggested that loss-of-function mutations in mildew resistance locus- o (*Mlo*) genes confer broad-spectrum resistance against pathogens, and hence, *Mlo* genes can confer an effective race-independent resistance in several crops [84,85]. Although the mechanism underlying *MLO*-based disease resistance remains unclear, some of their family members function by regulating fungal-penetration resistance by controlling vesicle fusion events [86]. Indeed, Qiu et al. (2015) generated transgenic *Rosa multiflora* expressing an antisense *RhMLO1* that exhibited enhanced resistance to powdery mildew [87]. Xiang et al. (2019) recently identified two *MLO* members, *RgMLO6* and *RlMLO7*, that are potential candidate genes that can induce resistance to powdery mildew in *Rosa* species [88]. Black spot disease is another major fungal disease caused by *Diplocarpon rosae* Wolf, a hemibiotrophic ascomycete. It is one of the most devastating and widespread fungal diseases of the rose and leads to huge economic losses [89]. Black and brown spots appear on leaves as the representative symptoms of the disease and, eventually, immature leaves become weak and fall from the plant. Defoliation decreases the photosynthetic area of plants, thus leading to a reduction in plant vibrance, thereby drastically lowering its ornamental value. A rice *chitinase* gene introduced into the rose-susceptible cultivar ‘Glad Tidings’ by particle bombardment conferred reduced susceptibility to black spot disease [90]. The black-spot-susceptible rose cultivars ‘Heckenzauber’ and ‘Pariser Charme’ were transformed with *chitinases*, *glucanases*, and *RIPs* from barley, and the transgenic plants exhibited a reduction of 40% in black spot diseases compared to that of the control [75]. Terefe-Ayana et al. (2011) reported the *Rdr1* locus as important for resistance to black spot diseases in roses, and this is useful for applications in rose breeding, including the use of genetic modification technology [56]. Recently, transcriptomic analyses of roses responding to the two fungal pathogens, *D. rosae* (black spot) and *P. pannosa* (powdery mildew), demonstrated that the genes related to common defense mechanisms were upregulated in black spot and that those related to photosynthesis and cell-wall modification were downregulated for powdery mildew, thus implying that distinct cellular responses are stimulated by different fungal pathogens, even in the same host [91]. *B. cinerea* is a notorious fungal pathogen responsible for gray mold disease in roses. *B. cinerea* conidia secretes phytotoxins and secondary metabolites during penetration into the host epidermis, ultimately causing host cell death [92]. Necrotic local lesions on petals are the major symptoms of *B. cinerea* infection in roses, and these infections rapidly develop during postharvest transport when the flowers are packed in boxes with a high relative humidity [93]. Petals are economically important organs, and when they are damaged, this causes large commercial losses in the rose industry. Despite its economic importance as a predominant pathogen, studies examining *B. cinerea* infections in roses are limited to the comparisons of pathogen behavior in model plants such as *Arabidopsis* [94]. Recently, transcriptomic analyses of rose petals infected by *B. cinerea* determined that *RcERF099,* a gene that encodes member of the AP2/ERF transcription factor family, is involved in the regulation of resistance against *B. cinerea* in rose flowers, and this finding can provide a stepping stone for further studies aiming to improve gray mold disease resistance in roses [95].

### 4.2. Chrysanthemum

The chrysanthemum (*Chrysanthemum morifolium*) is one of the most economically important and highly favored floricultural crops in terms of ornamental market value, and is used as a cut flower, pot flower, and garden plant [96]. It is a herbaceous perennial species belonging to *Asteraceae* and some of the family members, such as *Chrysanthemum morifolium* and *Chrysanthemum indicum,* have been widely used for medicinal tea and/or as materials in the cosmetic industry [97]. Chrysanthemums possess a higher ornamental value due to their abundant diversity in floral color and shape, which is the result of their large genome complexity and the allohexaploid background of the cultivated chrysanthemum [98]. Chrysanthemums are affected by a wide range of fungal diseases, including leaf spots, gray mold, rusts, and powdery mildew. A major aspect of chrysanthemum crop production relies on chemical control and this process exhibits only ephemeral benefits. The narrow genetic pool and complex hexaploid genome are major limitations for classical breeding to introduce disease-resistant traits. Thus, genetic transformation is a potential alternative to hasten the production of disease-resistant genotypes with improved targeted traits [99]. Leaf spots in chrysanthemums are caused by different fungi, including the *Alternaria* species, *Septoria chrysanthemi*, *Septoria chrysanthemella, Septoria obesa*, and *Cercospora chrysanthemi*. Symptoms appear on leaves as yellowish spots that gradually become dark brown and black, ultimately leading to premature leaf losses and consequent yield losses. Transgenic chrysanthemums, overexpressing *polygalacturonase-inhibiting protein* (*PGIP*) from *Prunus mumei*, exhibited improved resistance to Alternaria leaf spot [100]. Hairpins are pathogenic molecules encoded by *hrp* genes that can induce plant resistance by activating defense-signaling cascades. Overexpression of one such *hrp* gene, *hpaG_Xoo_,* conferred increased resistance to *Alternaria tenuissima* in chrysanthemums [101], and the introduction of the rice *chitinase* gene (*chiII*) in chrysanthemums cv. Snowball resulted in increased resistance to leaf spot caused by *Septoria obesa* [102]. Gray mold disease caused by *B. cinerea* is the predominant fungal disease in chrysanthemums. Leaves from infected plants possess brown water-soaked spots and the infected parts are covered with a grayish-brown, powdery mass of spores. Takatsu et al. (1999) produced transgenic chrysanthemum lines overexpressing a rice *chitinase* gene (*RCC2*), which showed enhanced resistance to gray mold disease [103]. Similarly, chrysanthemums cv. Shinba, overexpressing *N-methyl transferase* genes such as *CaXMT1*, *CaMXMT1*, and *CaDXMT1,* exhibited increased resistance to *B. cinerea*. Leaves from the transgenic lines produced 2.5-fold higher levels of salicylic acid compared to that of the wild type, thus leading to delayed occurrence of the disease and reduced disease index [104]. These *N-methyl transferases* methylate xanthosine derivatives can be used to yield caffeine that indirectly stimulates the defense network, thus inducing the systemic acquired resistance in the host plant [105]. White rust disease is caused by *Puccinia horiana Henn*. and is one of the most destructive fungal diseases in chrysanthemums. It spreads rapidly under humid conditions in greenhouses, ultimately resulting in considerable economic losses [106]. Symptoms typically appear on the adaxial leaf surface as pale green to yellow spots, that then exhibit raised buff or pinkish pustules. Stems, bracts, flower buds, and florets are infected in susceptible cultivars [107]. Transgenic chrysanthemums, overexpressing the *Cry1Ab* gene from *Bacillus thuringiensis* and a modified *sarcotoxin IA* gene from *Sarcophaga peregrine* (*msar*), exhibited a stronger resistance to white rust caused by *Puccinia horiana* and also exhibited *Helicoverpa armigera* resistance [108]. A recent study demonstrated that *CmWRKY15-1*, which encodes a WRKY transcription factor, plays a key role in the resistance to white rust caused by *P. horiana* by regulating the salicylic acid-mediated disease-resistance signaling pathway in chrysanthemums [109].

### 4.3. Petunia

*Petunia hybrida* is a popular ornamental hybrid with diverse floral colors and morphologies. It belongs to the *Solanaceae* family and is native to South America. Petunias possess a well-established record of being a model system for studying the molecular, genetic, and ecological factors that determine flower development [110,111] and can be affected by wilting, discoloration, and plant death. Verticillium wilt is caused by *Verticillium albo-atrum* that attacks the plant from the soil through a water-transport system. The infected leaves eventually turn brown and drop off from the plant. The petunia is infected by powdery mildew pathogens such as *Podosphaera xanthii*, *Golovinomyces orontii*, and *Oidium longipes*. Symptoms can be identified according to powdery white spores on the foliage [112]. Petunias are severely affected by *B. cinerea*, a foliar leaf pathogen that causes gray mold and leaf blight [113]. Transgenic *Petunia hybrida*, overexpressing the *endochitinase* gene from *Trichoderma harzianum*, alone or in combination with *osmotin*, exhibited resistance to *B. cinerea* [114]. Khan et al. (2011) developed transgenic petunia plants overexpressing the wasabi *defensin* (*WD*) gene from *Wasabia japonica* [115]. Expression of the AMP *defensin* increased resistance to *B. cinerea* in marker-free transgenic petunias. Similarly, transgenic *Petunia hybrida* plants, overexpressing the synthetic *chitinase* gene *Nakamura Ikuo Chitinase* (*NIC*) encoding Chitinase1 protein of *Rhizopus oligosporus*, exhibited enhanced resistance to *B. cinerea* [116]. Recently, reduced levels of *PhMLO1* expression achieved by introducing a *PhMLO1* RNAi construct resulted in improved resistance to powdery mildew in petunias. However, *PhMLO1* knockdown resulted in pleiotropic effects on petunia growth and development that may have a negative effect on the further development of strategies to create powdery mildew resistance by RNAi in petunias [117]. 

### 4.4. Lily

Lilies (*Lilium* spp.), cultivated as a flower crop and potted plant, are one of the most popular ornamental plants. Lilies are affected by major fungal diseases, including gray mold caused by *Botrytis elliptica*. Symptoms are characterized by oval or circular yellowish or red spots on the leaves. Infected floral buds become shriveled and distorted, and the plants can die, depending on the severity of the disease [118]. Bulb rot in lilies caused by *Fusarium oxysporum* produces the initial symptoms of the plant’s foliage yellowing and wilting. Even though the bulbs appear healthy, the roots develop a reddish-colored decay in the tips. The plants become stunted with yellow foliage and rotted scales. The transgenic *Lilium* oriental ‘Star Gazer’, developed by overexpressing the *RCH10* chitinase gene, conferred resistance to *B. cinerea* [119]. More recently, microRNA159 from *Lilium regale* (*lre-miR159*) has been reported to confer resistance to gray mold caused by *B. elliptica* in transgenic *Arabidopsis* by repressing the expression of its target gene *LrGAMYB* [120]. Additionally, overexpression of the *LsGRP1* gene encoding a class II glycine-rich protein from *Lilium*, conferred resistance to *B. cinerea* in *Arabidopsis*. The authors determined that *LsGRP1* plays a role as a pathogen-inducible switch to allow for activation of the immune response in the plant and to consequently induce fungal apoptosis [121]. Several candidate genes conferring resistance to fungal pathogens have been identified in lilies. Sun et al. (2016) reported that transgenic petunia plants, overexpressing the ATP-binding cassette transporter gene *LrABCF1* from *L. regale,* displayed increased resistance to *B. cinerea* and RNA viruses (cucumber mosaic virus and tobacco rattle virus) in petunias [122]. Similarly, the *glutathione-S-transferase* gene introduced by *L. regale* Wilson induced resistance to *F. oxysporum* in transgenic tobacco [123] and the overexpression of a 14-3-3 gene from *L. regale* Wilson conferred resistance to *Fusarium* wilt in transgenic tobacco [124]. Various genes induced in response to an *F. oxysporum* infection have been identified in *L. regale* Wilson [125,126,127] and the identified candidates serve as valuable resources to develop improved resistance to fungal pathogens in lily cultivars.

### 4.5. Other Ornamentals

Various ornamental plants, including the carnation, gladiolus, scented geraniums, African violets, and bentgrass, have been transformed to possess fungal disease resistance. Transgenic carnation harboring different combinations of *PR-1*, *osmotin*, or *chitinase* genes have been developed to induce resistance to *F. oxysporum* [128]. Resistance to *Fusarium* wilt was generated in transgenic carnation by transforming the bacterial *chitinase* gene from *Serratia marsecens* [129]. Later, the *jasmonate methyl transferase* gene was introduced into carnation for *Fusarium* resistance [130]. Transgenic gladiolus ‘Peter Pears’, developed by transforming with a synthetic antimicrobial peptide gene (*D4E1*), exhibited enhanced resistance to *F.oxysporum* [131]. Kamo et al. (2016) demonstrated that cell extracts from the transgenic gladiolus, overexpressing a fungal *exochitinase*, *endochitinase*, or a bacterial *chloroperoxidase*, could inhibit the growth of *F. oxysporum* [132]. The *Ace-AMP1* gene was transformed in scented geraniums to provide resistance to *B. cinerea*, and the expression level of the Ace-AMP1 protein was proportionally correlated with enhanced resistance to *Botrytis* sporulation [133]. *Glucanase* and *chitinase* genes were transformed into African violets to induce resistance to *F. oxysporum* and *Pythium* [134]. Transgenic glufosinate-resistant bentgrass (*Agrostis* spp.) plants, developed for herbicide resistance, exhibited increased resistance to fungal pathogens, including *Rhizoctonia solani* and *Sclerotinia homoeocarpa*, after a spraying with glufosinate herbicide, thus indicating that the nonselective herbicide glufosinate can be used to suppress some fungal pathogens in transgenic glufosinate-resistant bentgrasses [135]. SNP markers for linkage mapping and the transcripts that may be involved in *Botrytis* resistance have been recently identified in gerbera, and these findings may be useful for further studies of disease resistance [66]. Moreover, transcriptomic analyses performed in gerbera revealed candidate genes for resistance to powdery mildew, and these could provide valuable resources for developing powdery mildew-resistant gerbera cultivars [136]. Recent reports detailing the enhancement of fungal disease resistance in various ornamental plants are listed in Table 2.

## 5. Host-Induced Gene Silencing (HIGS) and Spray-Induced Gene Silencing (SIGS) Used to Control Fungal Pathogens

Originally, RNAi was identified as a biological gene-silencing mechanism that involves double-stranded RNA-mediated sequence-specific mRNA degradation, and this technique was subsequently applied to investigate gene function and to genetically engineer plants for beneficial purposes. Suppressing gene expression by silencing the target RNA plays a key role not only in exploring gene function but also in combating plant pathogens. RNAi-based pathogen control is a powerful alternative to synthetic fungicides for crop protection against fungal diseases. These strategies include HIGS and SIGS that are both environmental-friendly and can provide scope for controlling plant diseases [19,137,138,139]. HIGS functions are based on RNAi and involve the expression of sequence-specific double-stranded RNAs (dsRNAs) in the host plant to thereby silence its target genes [140]. The mechanism of HIGS includes the transformation of the host plant with either a dsRNA or a hairpin-structured dsRNA construct containing the targeted pathogen gene. The generation of small interfering RNAs (siRNAs), homologous to the target mRNA, leads to degradation of the target mRNAs within the pathogen, thus protecting the host plant from the pathogen [137,138]. HIGS has been extensively applied to various important crops to effectively control fungal pathogens. For example, HIGS has been successfully applied in tobacco plants to fight against the necrotrophic fungal pathogen *Sclerotinia sclerotiorum* by silencing the fungal *chitin synthase* (*chs*) gene. Transgenic tobacco lines exhibited a reduction in disease severity within 72 h, and this was correlated with the presence of detectable siRNAs in these plants. Thus, HIGS prevents the expression of endogenous fungal genes to thereby increase disease resistance [141]. Similarly, HIGS has been implemented in horticultural crops to control fungal pathogens, including *F. oxysporum* that causes Fusarium wilt in banana; *Verticillium dahliae* that causes verticillium wilt in tomato; and the oomycete pathogen *Phytophthera infestans* that causes late blight in potato [142,143,144]. Other crops that have been successfully employed for transgenic studies include wheat and barley to combat fungal pathogens such as *Puccinia* species; *Fusarium* species; *Blumeria graminis*; and *Magnaporthe oryzae* in rice [18,145,146,147,148,149,150,151]. Active application of HIGS to ornamental crops helps to produce varieties that are resistant to fungal pathogens.

In addition to the production of RNA-silencing molecules in planta as observed in HIGS, SIGS can also be used by spray-applied biopesticides to control the pathogen. Although HIGS is effective against various pathogens in different crops, it can only be applied to crops that are available for efficient transformation [152]. SIGS is a non-transformative approach for protecting plants from diseases. Externally synthesized dsRNAs targeting a specific pathogen gene are sprayed onto the surfaces of the host plant. This is followed by either the host plant absorbing the dsRNAs and the subsequent induction of RNAi machinery, where the dsRNAs or siRNAs are further transferred to fungal cells to modulate the fungal RNAi machinery, or by the fungal cells on the host plant directly uptaking the sprayed dsRNAs to trigger the fungal RNAi machinery directly. The RNAi machinery in the fungal pathogen silences the target gene to thereby enhance the resistance of the host plant to the disease [19,139,152]. SIGS has been employed in different crops and has been observed to be efficient in combating fungal diseases. Successful application of SIGS in controlling *B. cinerea* and its phytopathogenic fungus *Sclerotinia sclerotiorum* has been reported in canola plants [153]. The SIGS strategy was also applied to barley by spraying *CYP3*-dsRNAs onto barley leaves, thus conferring resistance to *Fusarium graminearum* after inoculation [19]. Application of SIGS in ornamental plants is still in its infancy, and only two studies have been reported in the *Dendrobium* orchid and rose. In the *Dendrobium hybrid*, direct application of crude bacterial extract, consisting of *MYB1*-dsRNAs, onto flower buds led to the suppression of *DhMyb1* expression, ultimately resulting in altered floral epidermal cell morphology [154]. Notably, Wang et al. (2016) revealed that gray mold caused by *B. cinerea* in roses can be controlled through the use of SIGS by spraying dsRNAs or small RNAs (sRNAs) targeting the *Bc-DCL1* and *Bc-DCL2* that are primarily responsible for the deliverable sRNA production, to allow for the silencing host immunity genes to function [152]. Furthermore, treatment with tobacco RNA extracts containing *Bc*-*DCL1*/*2*-sRNAs on rose flowers reduces gray mold disease symptoms caused by *B. cinerea*. Both HIGS and SIGS represent promising and sustainable future technologies that offer eco-friendly crop protection and efficient strategies to study fungal pathogen defense in ornamental plants.

RNAi-based biopesticides target pathogens with specificity and accuracy and have enormous potential as an alternative to chemical-based control methods. However, RNAi has its own limitations to overcome including the stable delivery of the topically applied dsRNA and the duration of its pathogen protection. Additionally, the generalized risk of RNAi, similar to any genetic modification technology of plants, has the potential to adversely impact human health and the environment. Unlike the other crop protection methods, evident risks to human health by RNAi technology appear minimal, as the uptake of these dsRNAs involves multiple barriers, ubiquitous presence of dsRNAs in animals, and general consumption of dsRNA from plant material [155]. Usually, unintended impacts of RNAi to the environment are case-specific and mostly in the species closely related to the target [156]. Since the RNAi specificity depends upon the sequence identity between siRNAs and mRNA targets, there is a risk of off-target effect which may lead to the silencing of other transcripts containing sufficient sequence identity [157]. This off-target may occur even in the host plant [158]. This can be minimized by careful design of dsRNAs on the target genes through identifying and avoiding the contagious matches to ensure the reduction in homology to off-target transcripts [159].

## 6. Conclusions and Future Prospects

**Figure 2:**
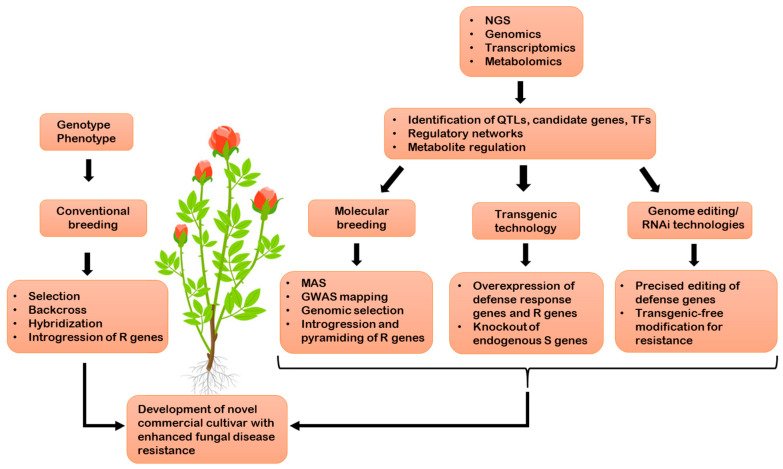
The comprehensive illustration of different strategies, integrating conventional and advanced breeding technologies to develop novel ornamental cultivars which possess enhanced fungal disease resistance traits.

Global commercial demand for ornamental crops is steadily rising. Approximately one third of the global ornamental market value comprises of cut flowers. Developing and introducing novel cultivars that possess the most desired ornamental attributes is vital in this ever-growing dynamic sector. Fungal diseases are considered to be the greatest threat to ornamental plants, contributing to huge losses in the host. Ornamental crops cannot tolerate fungal disease damage, as diseases directly affect the visual quality of cut flowers, an attribute that is critical for the esthetic value of attractive ornamental plants. Hence, the ultimate goal is to accelerate the development of novel cultivars that possess resistance to fungal diseases. Molecular breeding for fungal disease resistance in ornamental crops is lagging, primarily due to their complex genomes. Advances in next-generation sequencing (NGS) and multi-omics technologies have made it possible to obtain complete genome sequence information that can expedite the identification of a large number of molecular markers used for QTL mapping and GWAS. These data would be useful for exploring the complicated genetic mechanisms underlying fungal disease resistance. This enables the mining of candidate genes that are associated with disease-resistant traits and related pathways. The identified genes can be applied to genetic engineering to develop transgenic plants exhibiting enhanced resistance, a trait that constitutes a key component of disease management. Furthermore, genome editing tools such as CRISPR/Cas9 systems possess the potential to rapidly improve complex disease-resistance traits by editing either genes for susceptibility, or the genome of the pathogen itself [20]. However, the application of genome editing tools for fungal disease resistance in ornamental plants is still in its early stages. Due to the higher specificity, ease, and reproducibility of these tools, this breakthrough technology holds tremendous potential to enhance disease resistance in ornamental crops in the near future. Additionally, recent innovative strategies such as HIGS and SIGS have emerged as promising approaches to crop protection that are sustainable and environmentally friendly [140]. These approaches utilize the ability of dsRNA to silence the expression of target genes in plant pathogens. In particular, as SIGS does not require genetic transformation, it can be efficiently used for crop protection in ornamental plants. The combination of HIGS with the CRISPR/Cas9 approach will enhance fungal disease resistance in ornamental plants. A comprehensive illustration of breeding strategies to develop ornamental crops possessing improved fungal disease resistance is presented in Figure 2.

## Figures and Tables

**Table 1:** Turnover of the top 10 ornamental plants in the Royal FloraHolland auction in 2019.

S.No	Ornamental Plants Sold	Turnover (Million Euros)
1	Rose	696
2	Chrysanthemum	328
3	Phalaenopsis	460
4	Tulip	285
5	Gerbera	148
6	Lily	144
7	Kalanchoe	65
8	Anthurium	60
9	Potted rose	57
10	Lavender	20

**Table 2:** Recent advances in the genetic engineering of various ornamental crops for fungal disease resistance.

Crop	Gene	Disease Resistance	Reference
Rose (*Rosa hybrida*)	*Ace-AMP1*	powdery mildew *(Podosphaera pannosa)*	[82]
	rice *chitinase*	powdery mildew (*P. pannosa*)	[83]
	*RhMLO1, RgMLO6, RlMLO7*	powdery mildew *(P*. *pannosa)*	[87,88]
	rice *chitinase*	black spot *(Diplocarpon rosae*)	[90]
	*chitinases*, *glucanases*, and *RIPs*	black spot *(D*. *rosae*)	[75]
	*Rdr1*	black spot *(D*. *rosae*)	[56]
Chrysanthemum (*Chrysanthemum morifolium*)	*PGIP*	Alternaria leaf spot *(Septoria chrysanthemi)*	[100]
	*hairpinXoo*	leaf spot *(Alternaria tenuissima)*	[101]
	*chiII*	leaf spot *(Septoria obesa)*	[102]
	*RCC2*	gray mold *(B. cinerea)*	[103]
	*CaXMT1, CaMXMT1, CaDXMT1*	gray mold *(B. cinerea)*	[104]
	*Cry1Ab* and *sarcotoxin IA*	white rust *(P. horiana)*	[108]
	*CmWRKY15-1*	white rust	[109]
Petunia (*Petunia hybrida*)	*endochitinase* and *osmotin*	gray mold *(B. cinerea)*	[114]
	*WD (Wasabi defensin)*	gray mold *(B. cinerea)*	[115]
	*NIC (Nakamura Ikuo Chitinase)*	gray mold *(B. cinerea)*	[116]
Lily (*Lilium*)	*RCH10 chitinase*	gray mold (*B. cinerea*)	[119]
	*Ire-miR159*	gray mold *(B. elliptica)*	[120]
Carnation (*Dianthus caryophyllus*)	*PR-1, osmotin,* *chitinase*	Fusarium wilt *(F. oxysporum)*	[128]
	bacterial *chitinase*	Fusarium wilt *(F. oxysporum)*	[129]
	*jasmonate methyl* *transferase*	Fusarium wilt *(F. oxysporum)*	[130]
Gladioulus (*Gladiolus communis*)	*D4E1*	Fusarium wilt *(F.oxysporum)*	[131]
	Fungal *exochitinase, endochitinase,* bacterial *chloroperoxidase*	Fusarium wilt *(F. oxysporum)*	[132]
Geranium (*Pelargonium graveolens L. Herit.*)	*Ace-AMP1*	gray mold *(B. cinerea)*	[133]
African violets (*Saintpaulia ionantha*)	*glucanase* and *chitinase*	Fusarium and Pythium	[134]

## Data Availability

All the data is contained within the article.
